# From induction to conduction: how intrinsic transcriptional priming of extrinsic neuronal connectivity shapes neuronal identity

**DOI:** 10.1098/rsob.140144

**Published:** 2014-10-08

**Authors:** Jeffrey B. Russ, Julia A. Kaltschmidt

**Affiliations:** 1 Weill Cornell/Rockefeller University/Sloan Kettering Tri-Institutional MD-PhD Program, New York, NY 10065, USA; 2 Neuroscience Program, Weill Cornell Medical College, New York, NY 10065, USA; 3 Cell and Developmental Biology Program, Weill Cornell Medical College, New York, NY 10065, USA; 4 Developmental Biology Program, Sloan Kettering Institute, New York, NY 10065, USA

**Keywords:** subtype-specification, Ptf1a, Fezf2, transcription factors, neuronal identity, circuit formation

## Abstract

Every behaviour of an organism relies on an intricate and vastly diverse network of neurons whose identity and connectivity must be specified with extreme precision during development. Intrinsically, specification of neuronal identity depends heavily on the expression of powerful transcription factors that direct numerous features of neuronal identity, including especially properties of neuronal connectivity, such as dendritic morphology, axonal targeting or synaptic specificity, ultimately priming the neuron for incorporation into emerging circuitry. As the neuron's early connectivity is established, extrinsic signals from its pre- and postsynaptic partners feedback on the neuron to further refine its unique characteristics. As a result, disruption of one component of the circuitry during development can have vital consequences for the proper identity specification of its synaptic partners. Recent studies have begun to harness the power of various transcription factors that control neuronal cell fate, including those that specify a neuron's subtype-specific identity, seeking insight for future therapeutic strategies that aim to reconstitute damaged circuitry through neuronal reprogramming.

## 2. Introduction

The behaviours an organism employs to respond to its ever-changing environment depend on a highly intricate array of neuronal circuits, which in turn are composed of a vast assortment of neuronal subtypes. For this diversity of subtypes to assemble into functional circuits, it is imperative that the identity of each neuron be properly specified during nervous system development. In this review, we consider a neuron's subtype as the constellation of molecularly, morphologically and physiologically distinct characteristics that allow it to be distinguished from other neurons, including one particularly unique and functionally significant component of its identity, its connectivity. How then, is an undifferentiated neuronal precursor ultimately instructed to achieve its distinct identity and integrate appropriately into the emerging circuitry? Fundamental in this complex process is the interplay between a neuron's intrinsic transcriptional milieu and the extrinsic cues it encounters as it enters the surrounding network.

Internally, neuronal identity specification is controlled by an extensive hierarchy of transcription factors that act in concert to regulate neuronal development, many of which have been reviewed previously [1–6]. Fundamental in this transcriptional hierarchy is a class of subtype-specifying transcription factors that play a crucial role in guiding a neuron toward its terminal identity, often by selecting one fate over another competing fate, and by coordinating the expression of downstream gene batteries that direct the unique properties of a particular neuronal subtype [7,8]. These powerful transcription factors are both necessary and sufficient to induce key features of a subtype-specific identity, including the molecular expression patterns, morphology, electrophysiology and neurotransmitter status of a developing neuron.

It would be myopic to consider the internal effects of subtype-specifying transcription factors in isolation, however, as they also play a major role in determining a neuron's extracellular connectivity, dictating features such as dendritic morphology, axonal targeting and synaptic specificity. Once a neuron becomes incorporated into the surrounding circuitry, extrinsic signals from its pre- and postsynaptic partners then further refine its identity by regulating its transcription factor expression, neurotransmitter status, dendritic morphology or distinct synaptic protein profile. A neuron's connectivity thus lies at the intersection between the intrinsic and the extrinsic cues that together converge to determine a neuron's ultimate identity.

Two prototypical examples of subtype-specifying transcription factors, for which much is known about both their ability to specify neuronal identity as well as impact neuronal connectivity, are Fezf2 and Ptf1a. Fezf2 is expressed in layer V cortical pyramidal cells and is crucial for specifying not only the identity but also the unique connectivity of corticofugal projection neurons (CFuPNs) [9–12]. Ptf1a is more broadly expressed in different subpopulations of the nervous system and has been primarily implicated in specifying inhibitory interneurons of the spinal cord, cerebellum and retina [13–17]. Like Fezf2, recent work has demonstrated the role of Ptf1a in coordinating key aspects of neurite development and synaptic connectivity [18,19]. These two examples, therefore, serve to highlight the transcriptional basis of identity and connectivity specification, upon which the external neuronal network can then further refine neuronal identity.

In this review, we trace the process by which subtype-specifying transcription factor expression shapes neuronal connectivity and then provide examples of how early connectivity feeds back on neuronal identity. First, we survey how the subtype-specifying transcription factors begin to intrinsically establish neuronal identity, focusing primarily on Fezf2 and Ptf1a. We then outline studies that demonstrate how these factors prime a neuron's connectivity, identifying some known downstream mediators that assist in the process. We next discuss how early pre- and postsynaptic contacts of a developing neuron can refine its distinguishing properties, using well-characterized examples from neuromuscular and thalamocortical circuitry. Finally, we consider future applications of the subtype-specifying transcription factors in directing neuronal connectivity for circuit repair.

## 3. Early transcriptional regulators direct subtype identity specification

Invertebrate research has been instrumental in demonstrating the dependence of neuronal identity specification on the internal transcriptional milieu. As development of a neuronal precursor proceeds, the progressive expression of lineage-specific transcription factors leads the precursor through a sequence of regulatory states, culminating in the specification of its terminal neuronal fate [20,21]. A neuron's terminal fate typically arises from the actions of a master ‘terminal selector’, often selecting between two related terminal fates, which acts to induce a battery of terminal differentiation genes [7,8,22]. This terminal differentiation programme includes the receptors, cell-adhesion molecules and neurotransmitter machinery that provide the neuron with its unique subtype-specific properties [20,22,23].

In vertebrates, terminal neuronal fate is similarly governed by powerful transcription factors that act as selectors between related neuronal subtypes, inducing gene expression batteries that direct the acquisition of one terminal identity, while suppressing gene expression batteries of competing identities. For example, the basic motif-leucine zipper transcription factor Nrl acts in the retina to specify rod over cone photoreceptor identity. In *Nrl* mutant mice, rod precursors differentiate instead into cone-like photoreceptors, as determined by a shift to cone-like gene expression, morphology and electrophysiological properties [24,25]. Conversely, misexpression of Nrl in *Xenopus* retina is sufficient to increase the number of retinoblasts that differentiate into rods, at the expense of those that differentiate into cones [26]. As another example, the homeobox transcription factors Tlx1 and Tlx3 have been shown in the dorsal spinal cord to be necessary for the specification of an excitatory over an inhibitory interneuron fate. In mouse *Tlx1/3* mutant spinal cords, dorsal inhibitory interneurons are overproduced at the expense of excitatory interneurons, as indicated by the expanded expression of inhibitory transcription factors and neurotransmitter markers and a concomitant reduction of excitatory neurotransmitter markers [27]. Misexpression of Tlx3 in chick neural tube, on the other hand, is sufficient to suppress inhibitory transcription factors and GABAergic markers while upregulating glutamatergic markers [27]. Finally, in the ventral spinal cord, the LIM homeodomain transcription factor Lhx3 is necessary to specify a population of interneurons, called V2 interneurons, and in combination with Isl1, to specify motor neurons [28–30]. Misexpression of these transcription factors, on the other hand, is sufficient to ectopically upregulate V2 interneuron and motor neuron markers in the dorsal spinal cord [30], and their expression is also sufficient in mouse embryonic stem cells to induce a battery of motor neuron terminal differentiation genes [31].

**Figure 1.**
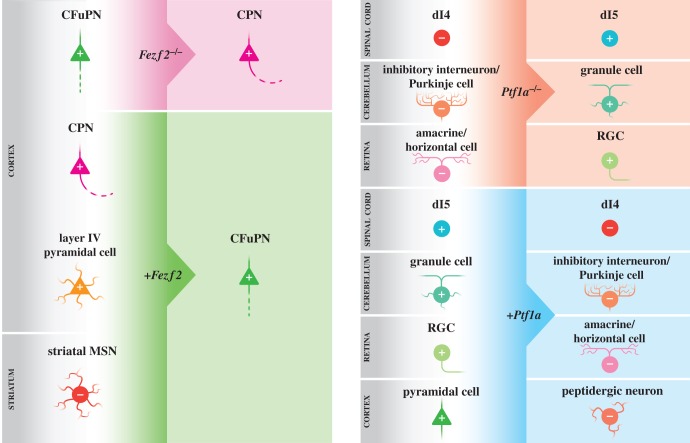
Fezf2 and Ptf1a are necessary and sufficient for a subtype-specific identity. Column 1 illustrates the role of Fezf2 in controlling CFuPN identity. In *Fezf2^−/−^* mice (row 1), CFuPNs primarily acquire a CPN identity, which causes these cells to project axons across the corpus callosum rather than to subcortical targets [10,32]. Misexpression of Fezf2 (*+Fezf2*) in CPNs or layer IV pyramidal cells in the cortex (rows 2 and 3), or in striatal medium spiny neurons (MSNs) (row 4), is sufficient to convert their identity to resemble that of CFuPNs, which includes changes in their molecular profile, neuronal morphology, and projection of axons to subcortical targets [11,12,33,34]. In the case of MSNs, this also includes a change in neurotransmitter status from inhibitory to excitatory [34]. Column 2 illustrates the role of Ptf1a in controlling an inhibitory neuronal identity in various regions of the CNS. Ptf1a is necessary for specifying the identity of dI4 interneurons in the spinal cord, inhibitory interneurons and Purkinje cells in the cerebellum, and amacrine and horizontal cells in the retina (rows 1–3). Without Ptf1a expression (*Ptf1a^−/−^*), these neurons adopt the features of their excitatory counterparts: dI5 cells in the spinal cord, granule cells in the cerebellum and retinal ganglion cells (RGCs) in the retina [13–17]. Misexpression of Ptf1a (*+Ptf1a*) in the developing spinal cord (row 4), cerebellum (row 5) or retina (row 6) is sufficient to promote an inhibitory interneuronal identity, causing dI5 cells to differentiate with dI4 properties in the spinal cord, granule cells to differentiate with inhibitory interneuron or Purkinje cell properties in the cerebellum, and RGCs to differentiate with amacrine and horizontal cell properties in the retina [35–40]. Misexpression of Ptf1a in cortical pyramidal cells (row 7) is sufficient to induce features of an inhibitory peptidergic identity, including an alteration in cellular morphology and neurotransmitter status [19]. ‘+’ indicates an excitatory neurotransmitter status, ‘−’ indicates an inhibitory neurotransmitter status.

Perhaps two of the most thoroughly investigated subtype-specifying transcription factors in the vertebrate nervous system, however, are Fezf2 and Ptf1a, and extensive research into their ability to direct aspects of neuronal identity and connectivity warrants a more comprehensive discussion of these examples. Like the examples described above, Fezf2 and Ptf1a are both necessary and sufficient for their respective neuronal subtypes, acting as a switch between developmentally related identities (figure 1). Knockout studies first demonstrated the necessity of Fezf2 for the specification of CFuPNs, particularly subcerebral projection neurons (SCPNs), in layer V of the cortex [9,10]. Without its expression, these neurons fail to acquire their typical layer V CFuPN identity, instead adopting a callosal projection neuron (CPN) or a layer VI corticothalamic projection neuron (CThPN) identity, as determined by changes to their molecular expression patterns, electrophysiological profile and axonal projections [10,32,41]. Furthermore, misexpression of Fezf2 in pyramidal cells of upper cortical layers alters their transcriptome to resemble CFuPNs, particularly SCPNs, inducing numerous downstream Fezf2-dependent markers and causing these cells to project axons to subcortical and subcerebral targets, as CFuPNs would [10,12,32]. Recently, Fezf2 has even been shown to be capable of redirecting neuronal identity in postmitotic cortical pyramidal cells of layer II/III and layer IV that have already acquired their layer specific identity, suggesting the power of Fezf2 to induce a CFuPN identity beyond a neuron's typical stage of developmental plasticity [11,33]. In these studies, misexpression of Fezf2 is sufficient to reprogramme the molecular expression, morphology, physiology and axonal targeting of these postmitotic neurons to resemble CFuPNs, while still maintaining them as viable, functional components of cortical circuitry.

The subtype-specifying transcription factor Ptf1a is more broadly expressed than Fezf2, acting in multiple regions of the central nervous system (CNS) to induce a number of subtype-specific identities, depending on the region of its expression. For example, in the spinal cord of *Ptf1a* mutants, inhibitory interneuron precursors in the dorsal horn switch their fate to become excitatory precursors, upregulating excitatory transcription factors and glutamatergic markers at the expense of inhibitory transcription factors and GABAergic markers [14]. Similarly, in the retina, Ptf1a is necessary to promote a horizontal or amacrine cell fate over a retinal ganglion cell fate [13,16], and in the cerebellum, Ptf1a is required for an inhibitory interneuron or Purkinje cell fate over a granule cell fate [15,17]. Ptf1a is also required to specify much of the cerebellar anlage, namely the inhibitory neuronal component, over a ventral pontine neuronal fate [42]. Not limited solely to inhibitory neurons, Ptf1a is necessary in the hindbrain to induce an excitatory inferior olivary climbing fibre fate over an excitatory pontine mossy fibre fate [43]. The sufficiency of Ptf1a has also been explored within the neural tube, retina and cerebellum by misexpressing it in the excitatory counterparts of its endogenous inhibitory precursors. Misexpression of Ptf1a in chick neural tube shows the reverse of the *Ptf1a* mouse mutant, suppressing the molecular markers and neurotransmitter status of dorsal excitatory interneurons while promoting those of inhibitory interneurons [35–37]. Furthermore, misexpression of Ptf1a in *Xenopus* or chick retina has a similar effect, inducing horizontal and amacrine fates over excitatory photoreceptor, bipolar and ganglionic neuronal fates [38,39]. Most recently, misexpression of Ptf1a in excitatory neuronal precursors of the cerebellar rhombic lip has demonstrated its sufficiency to promote an inhibitory molecular expression pattern and GABAergic neurotransmitter status in these cells [40]. Together, these studies demonstrate the significant role of Ptf1a in establishing the subtype-specific identity of a number of neuronal subpopulations.

**Figure 2.**
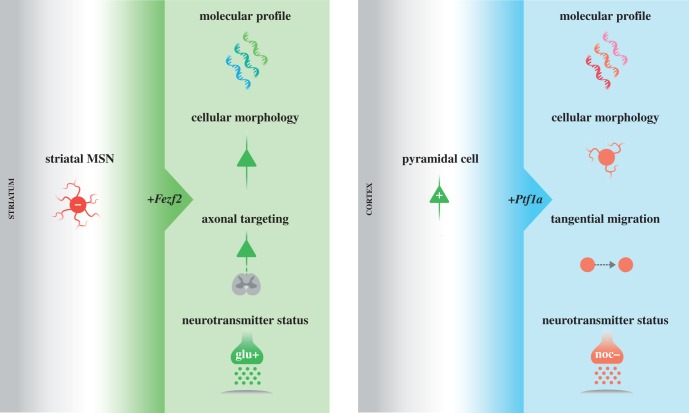
Misexpression of Fezf2 in striatal MSNs or of Ptf1a in cortical pyramidal cells cell-autonomously promotes subtype-specific features. Misexpression studies of Fezf2 in the striatum and Ptf1a in the cortex, regions of the CNS where these transcription factors are not endogenously expressed, demonstrate their sufficiency to cell-autonomously promote features of a CFuPN or inhibitory peptidergic interneuron, respectively. Column 1 illustrates how misexpression of Fezf2 (*+Fezf2*) induces MSN identity to resemble that of CFuPNs. From top to bottom, this involves alterations in the MSN molecular profile, a change in cellular morphology from a stellate to a pyramidal morphology, the induction of axonal targeting toward subcortical targets, such as the spinal cord, and a shift toward a glutamatergic neurotransmitter status [34]. Column 2 illustrates how misexpression of Ptf1a (*+Ptf1a*) induces pyramidal cell identity to resemble that of inhibitory peptidergic interneurons. From top to bottom, this involves alterations in the pyramidal cell molecular profile, a change in cellular morphology from a pyramidal to a more radial, branched morphology, adoption of a tangential migration pattern and the induction of a primarily nociceptinergic neurotransmitter status [15,19]. Plus sign ‘+’ indicates an excitatory neurotransmitter status, minus sign ‘−’ indicates an inhibitory neurotransmitter status. glu, glutamate; noc, nociceptin.

Beyond their endogenous function as an identity switch between developmentally related subtypes, another important property that attests to the potency of both Fezf2 and Ptf1a for neuronal subtype specification is their ability to cell-autonomously convert the identity of other neuron classes in distant regions of the CNS that normally exclude their expression (figures 1 and 2). For example, misexpression of Fezf2 in medium spiny neurons (MSNs) of the striatum is sufficient to alter their transcription factor expression, morphology, neurotransmitter status and axonal projection pattern to resemble those features in CFuPNs [34] (figure 2). The switch in MSNs from an inhibitory to an excitatory neurotransmitter status, in particular, is probably mediated directly by Fezf2, as Fezf2 has been shown to bind the promoters of both *Vglut1* and *Gad1*, activating the former and suppressing the latter [12]. These results imply that Fezf2 can act cell-autonomously to induce a CFuPN-like identity in MSNs, independent of the highly unfamiliar intra- and extracellular cues it encounters in the striatum. Similarly, studies of Ptf1a misexpression in developing cortical pyramidal cells have demonstrated its sufficiency to upregulate two direct molecular targets [44], as well as alter the migration and developmental trajectory of pyramidal cell precursors [15]. Use of RNA-seq to thoroughly characterize the extent to which Ptf1a misexpression alters the transcriptome of developing cortical pyramidal cells revealed that Ptf1a is capable of directly or indirectly inducing the expression of numerous inhibitory interneuronal genes in pyramidal cells, ultimately promoting an inhibitory peptidergic, primarily nociceptinergic, neurotransmitter status in these usually excitatory neurons [19] (figure 2). Moreover, these Ptf1a-dependent transformations lead to a shift in pyramidal cell morphology towards a more branched, radial shape, as might be expected for a Ptf1a-expressing interneuron [19] (figure 2).

One nuance of these studies is that while misexpression of Fezf2 in striatal MSNs and Ptf1a in cortical pyramidal cells can induce multiple dramatic changes to neuronal identity, a complete transformation to CFuPN or inhibitory peptidergic interneuron identities, respectively, is unlikely. Rather, the observed Fezf2- and Ptf1a-dependent changes probably result in a hybrid identity of ‘MSNs with CFuPN-like qualities' or ‘pyramidal cells with inhibitory peptidergic interneuron-like qualities’. Comparing Ptf1a-dependent alterations in the pyramidal cell transcriptome with a dataset of Ptf1a-dependent genes in the neural tube [45] revealed that Ptf1a-activated and suppressed genes in each region are, to a significant extent, non-overlapping [19]. These results highlight the role of other region-specific effects on gene expression for full identity specification, which is to be expected given that Ptf1a specifies different neuronal subtypes depending on its regional context. As future investigations determine the extent to which dramatically different precursors can be induced to transcriptionally and functionally mimic endogenous neuronal subtypes—a concept considered in greater depth below—these misexpression studies, nevertheless, demonstrate the potency of Fezf2 and Ptf1a to promote subtype-specific features even outside of their endogenous contexts.

Together, investigations of the subtype-specifying transcription factors, including Fezf2 and Ptf1a, illustrate the power of these factors to direct neuronal development by specifying the fate of one neuronal subtype over a competing fate. In the cases of Fezf2 and Ptf1a, they can even override a dramatically different internal transcriptional milieu and foreign extracellular cues, in order to cell-autonomously induce subtype-specific features in developmentally unrelated neuronal precursors. While the full array of such transcription factors continues to be elucidated, and the range of their capabilities remains to be seen, it is clear that this class of subtype-specifying transcription factors plays a fundamental role in shaping neuronal identity.

## 4. Identity and wiring: early transcriptional regulators instruct neuronal connectivity

As with many other features of a neuron's identity, its synaptic input and output are heavily dependent on the upstream actions of subtype-specifying transcription factors. These factors facilitate the neuron's ability to wire appropriately, whereupon it then encounters extracellular signals from its synaptic partners. While the molecular pathways that connect subtype-specifying transcription factors to their downstream mediators of neuronal connectivity are still being elucidated, the control these transcription factors impose on elements of circuit formation is becoming clear. For example, the misexpression of Fezf2 in layer IV pyramidal cells and MSNs of the striatum is sufficient to reshape their dendritic morphology, causing them to adopt a triangular CFuPN-like projection pattern [11,34]. In the case of layer IV pyramidal cells, misexpression of Fezf2 even results in the growth of a prominent apical dendrite, which layer IV neurons typically lack [11].

With regard to axonal development, Fezf2 is necessary for the downstream expression of Ctip2, a transcription factor required for the appropriate axonal architecture and subcortical targeting of CFuPNs [46]. Accordingly, Ctip2 is able to rescue the axonal targeting defects of *Fezf2^−/−^* neurons, restoring their projection to subcerebral targets, such as the spinal cord [32]. This supports the idea that the induction of Ctip2 by Fezf2 helps to establish the axonal properties that allow CFuPNs to reach their appropriate postsynaptic targets. However, even in some studies that do not find misexpression of Fezf2 to be sufficient to upregulate Ctip2, Fezf2 is still sufficient to induce CFuPN-like subcortical and subcerebral projection patterns in Fezf2-misexpressing cells, indicating that it uses other downstream mediators of axonal targeting in addition to Ctip2 [11,32,34]. One such mediator, the axon guidance receptor EphB1, was found to be directly activated by Fezf2 and is necessary for the proper ipsilateral descent of SCPN axons through the corticospinal tract (CST) [12]. Without EphB1 expression, SCPNs instead send aberrant contralateral projections across the anterior commissure [12].

Fezf2-dependent alterations in dendritic morphology and axonal projection patterns are also associated with changes to both electrophysiological output, as well as presynaptic input. In *Fezf2* mutant mice, CFuPNs of layer V adopt the characteristic spike frequency adaptation of CPNs, rather than a typical non-adapting or bursting pattern [32]. Conversely, misexpressing Fezf2 in layer IV pyramidal cells alters a number of their firing properties to resemble those of CFuPNs [11]. Moreover, Fezf2-misexpressing layer IV cells also receive altered neuronal input, losing their typical thalamocortical contacts and instead receiving input from layer II/III cells, as CFuPNs would [11]. These studies demonstrate how the subtype-specifying transcription factor Fezf2 is responsible for dictating many features of CFuPN connectivity, with clear functional consequences for the proper incorporation of CFuPNs into the surrounding circuitry.

Ptf1a has similar functions in priming a neuron's eventual circuit connectivity. As mentioned, misexpression of Ptf1a is sufficient to alter the dendritic projection pattern of cortical pyramidal cells, causing them to elaborate a more branched, radial array of neurites [19]. Also, with regard to synaptic development, Ptf1a is both necessary and sufficient for the expression of the cell-adhesion molecules Neph3/Kirrel2 and Nephrin/Nphs1 [19,44], whose invertebrate homologues have been implicated in synaptogenesis [47]. Along the same lines, in the mammalian spinal cord, Ptf1a is thought to be indirectly responsible for the synaptic specificity of a population of presynaptic inhibitory interneurons, called GABApre neurons. GABApre neurons rely on the synaptic binding protein NrCAM to mediate the adhesion interaction by which GABApre terminals bind with high density to their proprioceptive sensory terminal target [18]. Expression of *NrCAM* is reduced in the intermediate spinal cord of *Ptf1a* mutant mice (K. Kridsada and J. Kaltschmidt 2012, unpublished data), suggesting its dependence on Ptf1a expression in GABApre neurons, which reside in this region of the spinal cord [48].

Together, these studies suggest that a major role of subtype-specifying transcription factors, such as Fezf2 and Ptf1a, is to predispose developing neurons to adopt characteristics that affect their later incorporation into circuitry. Among other properties, these transcription factors impact dendritic morphology, axonal targeting, electrophysiological output and synaptic specificity, helping to shape a neuron's circuit connectivity.

## 5. Neuronal connectivity refines neuronal identity

Once the intrinsic transcriptional milieu helps to prime neuronal connectivity, consequent synaptic interactions of a developing neuron within its emerging neuronal network also provide critical extrinsic cues that further influence the neuron's acquisition of a particular identity. To this point, studies have demonstrated that the plasticity for reprogramming a postmitotic neuron via transcription factor misexpression diminishes after the first postnatal week [11,33], leading to speculation that the network gradually becomes more responsible for maintaining and fine-tuning neuronal identity as development proceeds [2].

Many distinguishing properties of neuronal subtype depend on contact with the postsynaptic target. These interactions are distinct from the target-derived cues that function more generally to support neuronal growth and survival or to regulate the mechanics of synapse assembly (reviewed in [49,50]). A well-established system for studying the influence of postsynaptic targets on neuronal identity formation is the neuromuscular circuitry. For example, in co-cultures of *Xenopus* muscle cells with spinal cord neurons, direct contact of the spinal neurons with target muscle cells suppresses the spinal neurons' ability to adopt a non-cholinergic neurotransmitter status [51]. These principles carry over to the mammalian nervous system, where muscle-derived trophic factors are critical for maintaining the expression of key regulators of neurotransmitter status in motor neurons of the facial nucleus [52,53]. Subtype specification of mammalian spinal motor neurons reveals an even more extensive role for target-derived cues. The peripheral expression of glial cell line-derived neurotrophic factor (GDNF) by the limb bud directs a subset of developing motor neurons to adopt a motor pool-specific molecular identity through the induction of the ETS transcription factor Pea3 [54,55]. Moreover, via the Pea3 pathway, GDNF indirectly regulates the position of these motor neurons in the spinal cord, their dendritic projection patterns and their monosynaptic innervation by sensory neuron terminals [54,56]. These studies suggest that contact with the postsynaptic target can modulate multiple aspects of a neuron's subtype-specific identity.

Presynaptic to the developing neuron, afferent-derived influences on neuronal identity have been described for thalamocortical projections onto layer IV spiny stellate neurons in the somatosensory cortex. For example, the afferent-derived molecules neuritin-1 and VGF are expressed in the thalamic neurons that project to the cortex and help specify the complex dendritic morphology of their postsynaptic spiny stellate neuron targets [57]. A recent study also showed that silencing incoming thalamocortical projections onto spiny stellate neurons in the mouse barrel cortex alters their molecular expression patterns, dendritic morphology and incorporation into functional barrel architecture [58].

Merging the concepts of post- and presynaptic influence on neuronal identity, the GABApre interneuron in the spinal cord affords an illustrative example of how one of its distinguishing characteristics, its synaptic protein profile, is modulated by both its postsynaptic target and its afferent input. The synaptic expression of the GABA-synthesizing enzyme GAD65 has been shown to distinguish the GABApre population from other populations of spinal interneurons [48,59]. Removal of BDNF–TrkB signalling between the postsynaptic sensory afferent terminal and the presynaptic GABApre neuron does not disrupt the formation of GABApre synapses onto sensory terminals, however it does prevent the hallmark accumulation of GAD65 in the GABApre terminals [48]. In addition, presynaptic input onto GABApre neurons from the CST plays a critical regulatory role for the synaptic expression of GAD65 in GABApre terminals. Developmental disruption of CST input, via cortical ischaemic injury, results in an over-accumulation of GAD65 in GABApre terminals, specifically on the side of the spinal cord affected by CST loss [60]. Thus, synaptic expression of GAD65, a defining feature of GABApre interneuron identity, is subject to proper wiring of both the postsynaptic and presynaptic partners of the GABApre interneuron.

## 6. Identity specification is malleable: neuronal programming and its implications for circuit repair

As the factors that control neuronal identity and connectivity have become better understood, much enthusiasm has been generated for harnessing these processes to repair neuronal circuits damaged by disease or injury. Central to this goal is the idea that neuronal identity is far more plastic than was once imagined. Originally, cellular differentiation was thought to descend through an ever-narrowing set of fate-specifying decisions until the cell reached its final identity. Waddington [61] originally conceived of this process as a marble that started at the top of a slope, representative of a cell's undifferentiated state, and then rolled down the slope through a series of grooves until it settled into one of the valleys at the bottom, symbolizing its fully differentiated identity. However, it has since become apparent that the directional limitations of the marble on the slope must be reconsidered, as it may be possible to push the marble back uphill or even transport it directly between valleys. Evidence is accumulating that under the right conditions, differentiated cells can return to an undifferentiated state and re-differentiate with new identities, or even transdifferentiate directly between identities (reviewed in [2,62]).

Increasing awareness of the plasticity of cellular identity has spurred a torrent of current research into *in vitro* and *in vivo* neuronal reprogramming. A variety of developmentally expressed transcription factors have begun to be explored for their ability to help generate specific neuronal subtypes, not only from other neurons, but also from closely related glia, or even more distant lineages, such as fibroblasts [34,63–66]. Often, these transcriptional cocktails include neurogenic transcription factors that direct a general neuronal identity, which are then supplemented with other subtype-inducing transcription factors. For example, the neurogenic transcription factors Brn2, Ascl1 and Myt1 are able to induce neuronal cells from fibroblasts [67]. When these three factors are accompanied by different sets of motor neuron-specific transcription factors, converted fibroblasts are induced to adopt the specific properties of motor neuronal identity, and even regionally distinct subtype identities [68,69]. Other studies have differentiated neurons with a midbrain dopaminergic (mDA) identity from fibroblasts, by combining various neurogenic factors with a variety of transcription factors that are critical for mDA fate specification, including FoxA2, Lmx1a and Nurr1 [64,70]. These initial studies are laying the groundwork for exciting new therapeutic strategies that aim to replace or repair damaged neurons in subtype-specific diseases, such as motor neurons in amyotrophic lateral sclerosis or mDA neurons in Parkinson's disease.

As promising strategies evolve to reprogramme neuronal identity, subtype-specifying transcription factors, such as Fezf2 and Ptf1a, which have been shown to cell-autonomously redirect neuronal subtype identity *in vivo*, may be ideal candidates to include in these studies. The ability of Fezf2 to reprogramme postmitotic neurons of other subtypes *in vivo* [11,33] is encouraging for studies of neuronal reprogramming through transdifferentiation, or direct conversion between subtypes. One advantage transdifferentiation is thought to have over induction from a more pluripotent precursor is decreased tumorigenic potential [67,70]. Use of existing neurons *in vivo* would also eliminate the need for transplantation. Furthermore, it has been proposed that *in vivo* transdifferentiation of closely related neighbouring neurons might reduce barriers toward establishing the correct wiring, as they may already share similar features with the desired neuronal subtype, which could help ease the transformation [2]. It will be intriguing to see whether any other subtype-specifying transcription factors are capable of postmitotic reprogramming and could be considered for transdifferentiation strategies that aim to reprogramme diseased circuit components.

However, as strategies for transcriptional neuronal reprogramming evolve, it is necessary to consider that the actions of any misexpressed transcription factor, including the subtype-specifying transcription factors, will be restricted by the epigenetic landscape of the neuron in which it is expressed. To this point, previous studies have demonstrated that the identity-specifying abilities of other transcription factors indeed rely on the cooperation of various mediators of chromatin modification. For example, the neurogenic transcription factor Pax6 associates with the chromatin remodelling Brg1-containing BAF complex to direct a neuronal over a glial fate [71]. Furthermore, acute knockout of the rod-specifying transcription factor Nrl in adult, postmitotic retinas caused only a partial transformation to a cone-like identity, and the observation that key rod-specific genes remained hypomethylated while key cone-specific genes remained hypermethylated suggests that full transformation may be prohibited by epigenetic mechanisms [72]. Ptf1a is not exempt from a dependency on epigenetic regulation. A recent study demonstrated that it can regulate distinct lineage-specific sets of genes in the neural tube versus the pancreas, which also uses Ptf1a, and that this discrepancy is linked to the tissue-specific chromatin arrangement of Ptf1a regulatory elements [73]. Thus, future studies of subtype-specifying transcription factor misexpression for neuronal reprogramming may benefit from the incorporation of other factors that facilitate epigenetic plasticity. Both the co-expression of transcriptional cofactors or the application of a histone deacetylase inhibitor to help prepare a favourable chromatin state are two potential strategies that have been examined, albeit with limited success [33,73]. Another promising strategy might be to include the use of microRNAs, which have been implicated in neuronal reprogramming [74], and act in part through the modulation of chromatin regulators such as the BAF complex [75]. Although a more nuanced understanding is required of how each of these techniques impacts neuronal identity specification, these strategies may play a promising cooperative role in future studies of neuronal reprogramming with subtype-specifying transcription factors.

Once the programming of a specific neuronal subtype becomes facile, the next major hurdle will be to ensure the proper connectivity of reprogrammed neurons. It is therefore convenient that a major downstream function of the subtype-specifying transcription factors is to prime aspects of a neuron's connectivity. Coupled with a better understanding of the extrinsic signals that shape identity, such as those imparted by the neuron's pre- and postsynaptic partners, our ever-improving ability to manipulate each of these variables will eventually allow us to control the subtleties of neuronal identity and connectivity with increasing precision. While the therapeutic uses of neuronal reprogramming to regenerate damaged microcircuitry are still in their infancy, there is no question that transcription factors that can cell-autonomously instruct neuronal subtype identity and connectivity will continue to play a fundamental role in the study of reprogramming for circuit repair.

## 7. Conclusion

While a functional organism requires the proper development of each and every cell, the precise coordination of both identity specification and incorporation into the surrounding tissue is nowhere more essential than for neurons within the nervous system. Each of the innumerable neuronal subtypes must first be generated, and then each must wire appropriately with its synaptic partners, in order to establish a network capable of forming memories, planning and executing actions, or generating emotions. A critical event that regulates this process during early neuronal development is the expression of subtype-specifying transcription factors, such as Fezf2 or Ptf1a. These transcription factors are powerful regulators of many aspects of neuronal identity, and are both necessary and sufficient to cell-autonomously confer subtype-specific features to developing neurons. One major function of the subtype-specifying transcription factors is to prime various elements of a neuron's connectivity, such as its dendritic morphology, axonal targeting and synaptic specificity. As these features help the neuron become incorporated into early neuronal networks, synaptic contacts from pre- and postsynaptic partners help to refine its identity, by further regulating its molecular expression pattern, dendritic morphology, neurotransmitter status or synaptic protein profile. Replacing damaged microcircuits is one of the eventual therapeutic goals of studies that aim to use subtype-specifying transcription factors for neuronal reprogramming. As our understanding of the interplay between intrinsic transcriptional control and extrinsic network control over neuronal identity becomes more sophisticated, a future of successful circuit repair through neuronal reprogramming appears ever more within reach.
